# Significance of Brownian Motion for Nanoparticle and Virus Capture in Nanocellulose-Based Filter Paper

**DOI:** 10.3390/membranes8040090

**Published:** 2018-10-05

**Authors:** Olof Gustafsson, Simon Gustafsson, Levon Manukyan, Albert Mihranyan

**Affiliations:** Division of Nanotechnology and Functional Materials, Department of Engineering Sciences, Uppsala University, Box 534 SE-75121 Uppsala, Sweden; olof.gustafsson@kemi.uu.se (O.G.); simon.gustafsson@angstrom.uu.se (S.G.); levon.manukyan@angstrom.uu.se (L.M.)

**Keywords:** virus removal filtration, Péclet number, nanocellulose, hydrodynamic constraint, convective capture, diffusion

## Abstract

Pressure-dependent breakthrough of nanobioparticles in filtration was observed and it was related to depend on both convective forces due to flow and diffusion as a result of Brownian motion. The aim of this work was to investigate the significance of Brownian motion on nanoparticle and virus capture in a nanocellulose-based virus removal filter paper through theoretical modeling and filtration experiments. Local flow velocities in the pores of the filter paper were modeled through two different approaches (i.e., with the Hagen–Poiseuille equation) and by evaluating the superficial linear flow velocity through the filter. Simulations by solving the Langevin equation for 5 nm gold particles and 28 nm ΦX174 bacteriophages showed that hydrodynamic constraint is favored for larger particles. Filtration of gold nanoparticles showed no difference in retention for the investigated fluxes, as predicted by the modeling of local flow velocities. Filtration of ΦX174 bacteriophages exhibited a higher retention at higher filtration pressure, which was predicted to some extent by the Hagen–Poiseuille equation but not by evaluation of the superficial linear velocity. In all, the hydrodynamic theory was shown able to explain some of the observations during filtration.

## 1. Introduction

Filtration is an essential step in many industrial processes. As the size of the particles is decreased, it becomes technologically challenging to achieve high separation efficiencies. This is especially true for particles featured with nano dimensions (e.g., proteins, viruses, bacteriophages, inorganic nanoparticles) since their behavior during filtration is different from that on the macroscale. Probably the most extreme case of filtration of nanoparticles is that of viruses, which are capable of replicating under favorable conditions in biological organisms. Thus, virus removal filtration is regarded as the most critical steps in the manufacturing of protein-based pharmaceutics to ensure virus safety [1]. Although several industrial size-exclusion virus removal filters are currently available, virus breakthrough in filters with pores that are nominally smaller pores than the virus size have been described depending on the process conditions [2,3,4,5]. One parameter that was shown to affect the virus breakthrough is the applied trans-membrane pressure or pressure release. A decrease in virus retention capacity was observed in some membranes after the pressure release, which was ascribed to the migration of previously captured viruses. See Supporting Information for more information on previous observations of pressure-dependence in the removal of bioparticles in filtration [2,3,4,5,6,7].

Small particles suspended in a liquid will be subject to random motion caused by collisions with the molecules in the liquid, i.e., Brownian motion. Brownian motion is a slow process on the macroscopic scale, but it is a much faster process on the nanometer scale. For example, a 28 nm ΦX174 bacteriophage [8] placed at the center of a 100 nm wide pore could encounter the pore wall through Brownian motion in 4 × 10^−5^ s at zero-flow conditions. Thus, with virus filtration involving interactions between viruses and pores on the scale of nanometers, the influence of the Brownian motion on virus behavior during filtration cannot be neglected.

Attempts of modeling the underlying physical mechanisms for the observed pressure dependent retention of particles in virus filters were carried out. Trilisky and Lenhoff [9] studied the flow-dependent entrapment of bioparticles in a porous bead chromatography medium and suggested a mechanism for particle retention. The proposed mechanism thus relates convective and diffusive forces on the motion of particles throughout the porous structure of the medium. The authors envisioned particle entrapment to occur at pore constrictions smaller than the diameter of the particle, where the particle was only able to escape entrapment if diffusive forces could overcome convective forces. As a result, at low flow velocities, particles were expected to avoid entrapment by diffusion and thus, higher throughput of particles was expected as confirmed in constant flow filtrations of the Ad5 virus [9]. Yamamoto et al. [10] presented an extensive study on the pressure dependent removal of viruses in Planova™ filters.

In a flowing fluid, the particle movement is governed by two components (i.e., convective forces), due to viscous drag and diffusion due to random Brownian motion. The Brownian motion of particles in a flowing fluid can be described by the Langevin equation [11]. Extensive discussion of the theory is presented in the Supporting Information [10,11,12,13,14,15,16,17,18].

In the present work, the effect of Brownian motion on nanoparticle and virus capture is explored further in non-woven virus removal filter paper. The nanocellulose-based virus removal filter paper evaluated in this study is a filter with high porosity, tunable pore-size distribution and a stratified structure produced from wet-laid nanocellulose fibers [19,20]. The wet strength of the filter paper can be increased by cross-linking the cellulose nanofibers with citric acid to allow for higher filtration pressure gradients [21]. Virus removal capacity of the filter paper was further reported for various model viruses, including a log_10_ reduction rate (LRV) >5 for the worst-case small size model parvovirus, i.e., minute virus of mice (MVM; 18–20 nm) [19,22,23,24]. Protein throughput of the filter paper showed high recovery and inertness for bovine serum albumin (BSA, MW 66 kDa) and lysozyme (14 kDa), whereas γ-globulin (MW > 175 kDa) was not able to pass through the filter due to its large size [25]. High virus removal rates and high protein recovery in combination with a simple manufacturing process from a readily available, sustainable material makes studying the virus removal mechanisms in these filters of interest [26].

Thus, the aim of this work was to investigate the effect of Brownian motion on particle capture in the nanocellulose-based filter paper through theoretical modeling of nanoparticle and virus motion and their local flow velocities as well as through experimental filtration at different fluxes and pressure gradients.

## 2. Materials and Methods

### 2.1. Materials

*Cladophora* algae cellulose was obtained from FMC BioPolymer (Newark, DE, USA) (batch G3828-112). 5 nm gold particles in 0.1 mM phosphate buffered saline (PBS) aqueous solution (752568-25ML, lot numbers MKBW8968V and MKBV6029V), phosphate buffered saline (P4417) and sodium chloride (S5886) were obtained from Sigma-Aldrich (Saint Louis, MO, USA). Escherichia coli bacteriophage ΦX174 (ATCC^®®^ 13706™) and Escherichia coli (Migula) Castellani and Chalmers (*E. coli*) (ATCC^®®^ 13706-B1™) were obtained from ATCC (Manassas, VA, USA). Yeast extract (212750), tryptone (211699) and agar (214530) were obtained from BD (Franklin Lakes, NJ, USA).

### 2.2. Preparation of Filter Paper

A 0.1 wt.% dispersion of Cladophora cellulose was prepared by adding 1 g of cellulose in 1 L of deionized water under stirring. The dispersion was then run twice in succession through a 200 µm and a 100 µm hole sized chamber at 1800 bar, using an LM20 Microfluidizer. 50 mL of the dispersed solution was then further diluted in 200 mL of deionized water and then drained over a nylon filter membrane (Durapore, 0.65 µm DVPP, Merck Millipore, Burlington, MA, USA) fitted in a funnel using a vacuum. The resulting wet cellulose mass was then dried at 170 °C using a hot-press (Rheinstern, Mainz, Germany).

### 2.3. Thickness Evaluation

The thickness of the manufactured filter papers was evaluated using a Mitutoyo Absolute digital caliper (ID-C150XB) with a precision of 1 µm. The thickness was measured on five different filters at five different positions on each filter. The results presented in Supporting Information Table S1.

### 2.4. Filtration of Gold Nanoparticles

Filtration of 5 nm gold particles through nanocellulose filter papers was carried out in constant flow mode using an NE-1010 syringe pump (New Era Pump Systems, Farmingdale, NY, USA). Filtrations of gold particles were carried out at two different flux settings; 0.1 mL/min and 0.5 mL/min. The permeate solution was collected in fractions during the experiment and the real flux was monitored using a digital scale (Mettler Toledo, Columbus, OH, USA, MS1602TS). The collected fractions were analyzed using a TECAN M200 spectrophotometer. The absorbance was measured between 460–600 nm and the background absorption of the PBS solution was subtracted from the measurements. The area under the curve (*AUC*) was calculated for the absorption peak by integration over the measured wavelengths using MATLAB.

The particle removal efficiency is described by the logarithmic reduction value (LRV) and is calculated using Equation (1).

(1) $$\begin{array}{c} LRV=log_{10}\frac{AUC_{feed}}{AUC_{permeate}} \end{array}$$

*AUC_feed_* is the area under the curve for the feed solution and *AUC_permeate_* is the area under the curve for the permeate solution. See Supporting Information for full procedure.

### 2.5. Filtration of ΦX174 Bacteriophages

Filtration of ΦX174 bacteriophages was carried out using an Advantech KST 47 filter holder. The nanocellulose filter papers were fitted in the holder using a Munktell General Purpose Filter Paper as a mechanical support. The filters were wetted with Luria–Bertani (LB) medium prior to filtration. Filtrations were carried out at two different overhead pressures; i.e., 1 bar or 3 bar. For each pressure two different volumes passed through the filters; i.e., 20 mL or 60 mL. The permeate solutions were collected and for filtrations of the larger volume, permeate was collected in two fractions of 30 mL each. The time for each filtration was monitored to determine the average flux during filtration. See Supporting Information for the full procedure and how to calculate the LRV for the bacteriophages.

### 2.6. Theoretical Modeling of Hydrodynamic Velocity and Brownian Motion

Code was developed in MATLAB to solve Equations (S9) and (S10) (see Supporting Information) for particle velocity and position [10,17]. The total time in the simulations was set to 1 × 10^−4^ s and each particle could take 300 steps during this time. All particles were approximated as hard spheres and the physical characteristics of the particles as well as other physical parameters used in the simulations can be found in the Supporting Information [8,27]. The flow in all simulations was constant and directed in the negative y-direction. Flow velocities were varied from 1 × 10^−5^ m/s to 5 × 10^−2^ m/s, and simulations with a flow velocity of 0 m/s were used as a reference. An artificial semipermeable wall was set up at y = 0 nm to simulate a filter surface, where particles are retained by pores of smaller size than the particles. Particles were unable to pass through the wall, but the flow was held constant and unaffected by the wall. All simulations began with placing the particles in contact with the wall at (0,0). No interaction between particles nor pore wall was considered.

## 3. Results and Discussion

**Figure 1:**
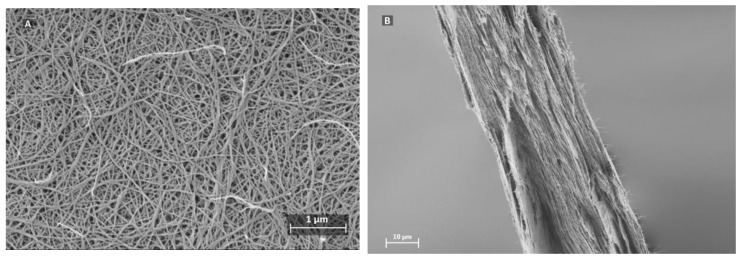
Scanning electron microscope (SEM) images of (**A**) top and (**B**) cross-section of the nanocellulose filter, featuring non-woven, and layered structure.

In order to investigate the effect of Brownian motion on particle capture, two approaches were taken; i.e., first finding the flow velocity where convective forces overcome Brownian motion and then comparing with the local flow velocity in the nanocellulose-based filter paper. Figure 1 presents scanning electron microscope (SEM) images of the filter paper. The non-woven structure can be observed in Figure 1a, where the random fiber mesh is visible from the top view. Cross-section images in Figure 1b reveal a stratified layered structure composed of nanocellulose-sheets stacked that is self-assembled and produced in the same manner as regular filter paper.

### 3.1. Theoretical Modeling of Hydrodynamic Velocity and Brownian Motion

**Figure 2:**
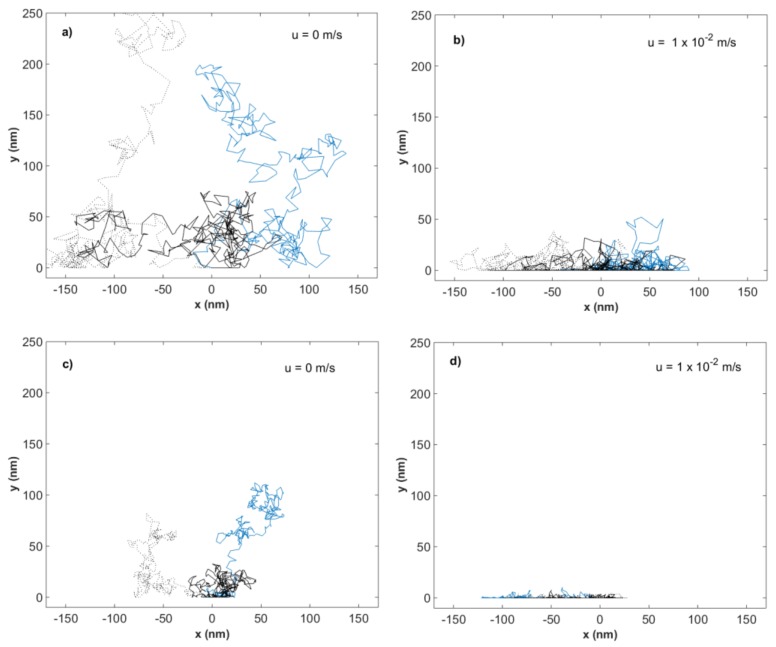
Simulated particle trajectories for 5 nm gold particles in water at flow velocities (**a**) 0 m/s and (**b**) 1 × 10^−2^ m/s and 28 nm ΦX174 bacteriophages in water at flow velocities (**c**) 0 m/s and (**d**) 1 × 10^−2^ m/s. Each line represents one particle trajectory.

The effect of different flow velocities on the Brownian motion of 5 nm gold particles and 28 nm ΦX174 bacteriophages was examined through simulations in MATLAB (R2016b), using the same approach as described by Satoh [17] and Yamamoto et al. [10] Figure 2a,b show results from the simulations of flow velocity effect on the Brownian motion of 5 nm gold particles for flow velocities 0 and 1 × 10^−2^ m/s. Figure 2c,d show results from the simulations of flow velocity effect on the Brownian motion of 28 nm ΦX174 bacteriophages for flow velocities 0 and 1 × 10^−2^ m/s. The results from simulations of both gold particles and bacteriophages at additional flow velocities can be found in the Supporting Information, Figures S2 and S3. Each plotted line indicates the trajectory of an individual particle during the time set in the simulations.

As seen from Figure 2, the magnitude of the Brownian motion at zero flow decreases with increased particle size. The Brownian motion is greatest for the smaller gold particles, while the larger ΦX174 bacteriophages show the shortest net distance traveled. The effect of hydrodynamic forces on the motion in the y-direction can be seen for both gold particles and ΦX174 bacteriophages as flow velocity is increased. The constraint is larger for the bacteriophages, compared to gold particles, at an equal given flow velocity. The movement in the x-direction remains unhindered throughout the simulations, which can be expected as there is no x-component of the flow.

### 3.2. Evaluation of the Péclet Number and Critical Flow Velocity for Particle Constraint

The results of the simulations suggest that at flow velocities of 1 × 10^−2^ m/s or higher, the hydrodynamic constraint is noticeable in the simulations for both tested particle types. As seen in the Supporting Information, Figures S2 and S3, at flow velocities of 1 × 10^−3^ m/s or lower, Brownian motion becomes prevalent and the mean traveled distance is dependent on particle size. Thus, the concept of a critical flow velocity, *u_cr_*, can be introduced and quantified by the Péclet number (*Pe*), which is further discussed in the Supporting Information [9].

The Péclet number relates the convective forces exerted on a particle from the flow to the diffusive forces caused by Brownian motion. *Pe* << 1 means that Brownian motion is the dominant force acting on the motion of a particle. However, if *Pe* >> 1 convection is the governing force. Figure S4 in the Supporting Information illustrates the relation between the Péclet number and the flow velocity for 5 nm gold particles and 28 nm ΦX174 bacteriophages.

Defining the critical velocity as the flow velocity where *Pe* = 1, the critical flow velocity, *u_cr_*, is 5.2 × 10^−4^ m/s for the ΦX174 bacteriophages and 1.7 × 10^−2^ m/s for the 5 nm gold particles. Thus, the hydrodynamic constraint of particles is possible at flow velocities above these critical values. For the gold particles, the Péclet number at the flow velocity 1 × 10^−2^ m/s is close to 1, indicating a stronger contribution from Brownian motion as compared to the ΦX174 bacteriophages at the same flow velocities, where *Pe* >> 1.

### 3.3. Modeling of Local Flow Velocities

Assuming a capillary pore geometry, the flow in a pore can be calculated using the Hagen–Poiseuille equation, which relates the flow in a cylindrical capillary to the pressure drop across the capillary. The Hagen–Poiseuille equation is shown in Equation (2) [28].

(2) $$Q=\frac{\Delta P\pi R^{4}}{8\eta L}$$

*Q* is the volumetric flow, *ΔP* is the pressure difference across the capillary, *R* is the radius of the capillary, *η* is the dynamic viscosity of the fluid and *L* is the length of the capillary. More information is available in Supporting Information [29].

**Figure 3:**
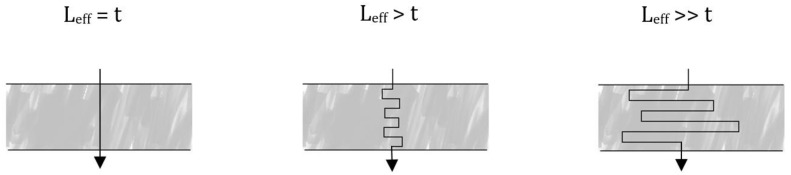
Illustration of the concept of effective pore length (*L_eff_*) relating it to the filter thickness (*t*) for different degrees of tortuosity.

Assuming a filter with an interconnected continuous pore structure and a constant thickness, the concept of an effective pore length, *L_eff_*, can be introduced. *L_eff_* is the length of a straight, cylindrical pore corresponding to a tortuous pore of length *L* stretching throughout the filter structure. As seen in Figure 3a higher degree of tortuosity will result in pores with a greater effective length.

**Figure 4:**
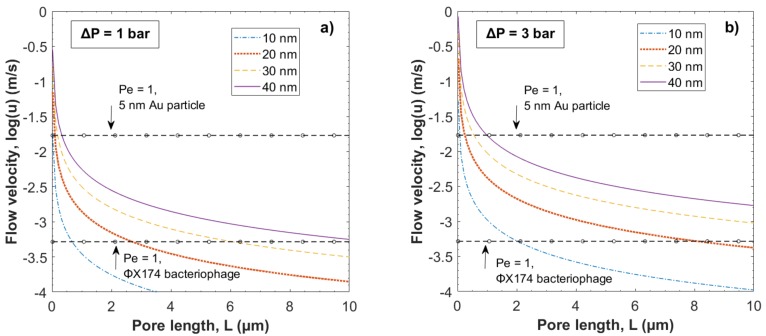
Local flow velocity, log *u*, as a function of pore length *L* at four different pore widths, i.e., 10, 20, 30 and 40 nm and pressures (**a**) 1 bar and (**b**) 3 bar. Dashed lines with circles indicate *Pe* = 1, i.e., *u* = *u_cr_* for the 5 nm gold particles and the 28 nm ΦX174 bacteriophages.

In Figure 4 the local flow velocity in cylindrical pores of ranging widths, i.e., 10, 20, 30 and 40 nm, was calculated using Equation (2) and plotted as a function of pore length *L* at two different *ΔP*; i.e., 1 and 3 bar.

According to the Bernoulli principle [6], the flow velocity profile through a pore of uniform radius will be the same throughout the pore in the case of laminar flow; i.e., *Re* < 2300. Thus, Figure 4 suggests that for pores of equal width and equal pressure gradient, the flow velocity will be lower for pores of greater length *L*. Thus, a critical pore length *L_cr_* can be defined, which states the maximum pore length where hydrodynamic constraint of a particle at given size, weight and flow velocity is expected. *L_cr_* is given by the intercept between the plotted curves and the dashed lines indicating *Pe* = 1 in Figure 4. For ΦX174 bacteriophages *L_cr_* is <0.68 µm for 10 nm pore width and <10.8 µm for 40 nm respectively.

**Figure 5:**
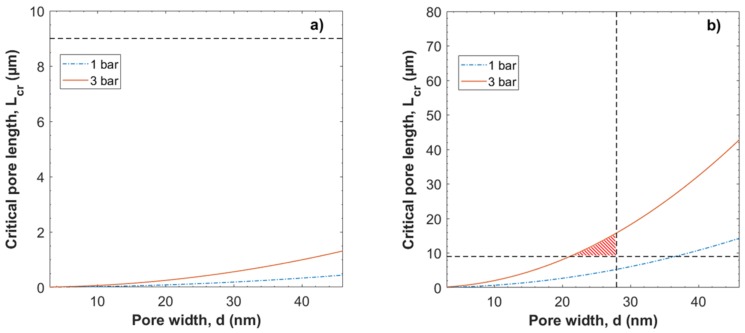
Critical pore length *L* as a function of pore width *d*, i.e., *u* = *u_cr_*, for (**a**) 5 nm gold particles and (**b**) ΦX174 bacteriophages at pressures 1 and 3 bar. Dashed lines at *L_cr_* = 9 µm and *d* = 28 nm indicate filter thickness and diameter of the ΦX174 bacteriophage respectively; Highlighted areas in (**b**) indicate pore lengths and widths where hydrodynamic constraint is possible.

In Figure 5, the critical pore length *L_cr_* is presented across pore widths between 3–46 nm at pressures 1 and 3 bar. Figure 5 suggests that if the effective pore length increases, e.g., through an increase in the tortuosity or the thickness of the filter, to a length *L* > *L_cr_* when the flow velocity *u* < *u_cr_*, will result in Brownian forces being the dominate factor for particle motion. As a result, at pore lengths *L* > *L*_cr_, hydrodynamic constraint of particles is not expected to occur.

For the smaller gold nanoparticles, *L_cr_* greater than the filter thickness (i.e., 9 (σ = 1) µm) (see Supporting Information), even at 3 bar, which is the theoretical minimum pore length for intact pores throughout the filter structure. Thus, hydrodynamic constraint is not expected for gold particles at pressures 1 and 3 bar according to this model.

For the ΦX174 bacteriophages, *L_cr_* is greater than the filter thickness at a pressure of 3 bar for pores of width ≤28 nm; i.e., pores of a width equal to or smaller than the diameter of the ΦX174 bacteriophage. From a size-exclusion perspective, pores of a width larger than the diameter of the ΦX174 bacteriophage are not expected to contribute to capturing and removal of the ΦX174 bacteriophages. Thus, hydrodynamic constraint is possible only at the higher pressure of 3 bar at pore lengths and widths indicated by the highlighted area in Figure 5b.

According to the hydrodynamic theory of virus capture, free Brownian motion favors virus breakthrough. There is, however, no experimental evidence that more virus breakthrough is observed when the thickness of the filter or the pore tortuosity is increased. Quite the opposite, it was previously shown that the filter breakthrough occurs when the filter thickness is decreased [19]. The latter highlights the limitations of the hydrodynamic theory of virus capture and its ability to predict virus breakthrough.

A different approach to estimate the local flow velocities in pores of different width was described by Trilisky and Lenhoff [9]. Assuming the flow velocity, u, in a pore being proportional to the square of the pore width [6], *u* can be approximately related to the superficial linear velocity, us, through a porous material according to Equation (3) [9].

(3) $$u\approx \frac{u_{s}}{\varepsilon }{\left(\frac{d}{d_{m}}\right)}^{2}$$

where *ε* is the porosity of the material, *d* is the width of the pore and *d_m_* is the mean pore width. The mean pore width *d_m_* was set to 23 nm which is the pore mode obtained for nitrogen gas sorption measurement of the non-woven filter used in the next section for evaluating the simulations [30]. The pore size distribution can be seen in the Supporting information Figure S7.

**Figure 6:**
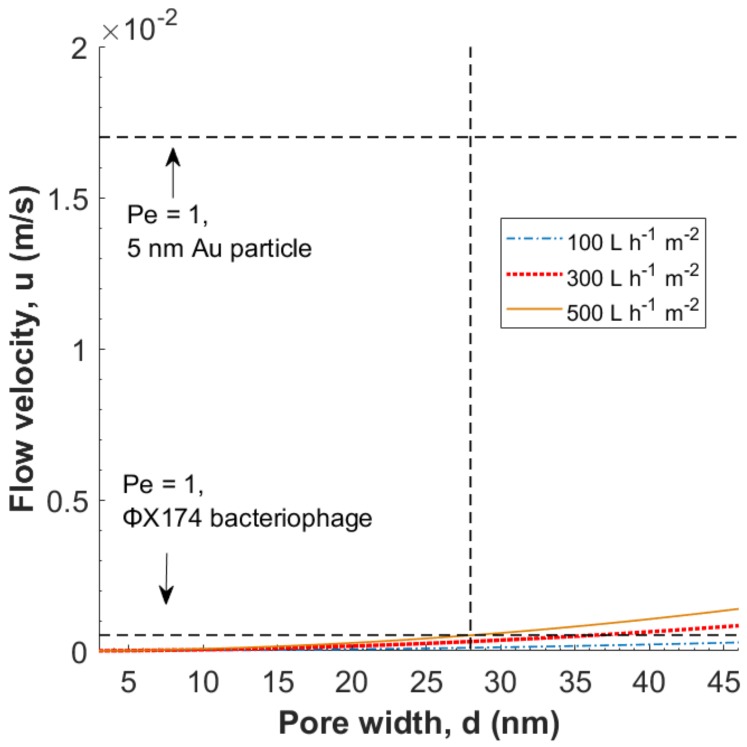
Local flow velocity *u* in pores of width 3–46 nm and *d_m_* = 23 nm, at fluxes 100, 300 and 500 L h^−1^ m^−2^. Figure constructed using Equation (3). Horizontal dashed lines indicate *Pe* = 1, i.e., *u* = *u_cr_*, for 5 nm gold particles and ΦX174 bacteriophages respectively. The vertical dashed line at *d* = 28 nm highlights a pore width equal to the diameter of the ΦX174 bacteriophage.

In Figure 6, the local flow velocity in pores of widths 3–46 nm with *d_m_* = 23 nm is presented for different superficial linear velocities (fluxes); i.e., 100, 300 and 500 L h^−1^ m^−2^. The porosity (*ε*) was set to 40%, as reported previously by us [20].

**Figure 7:**
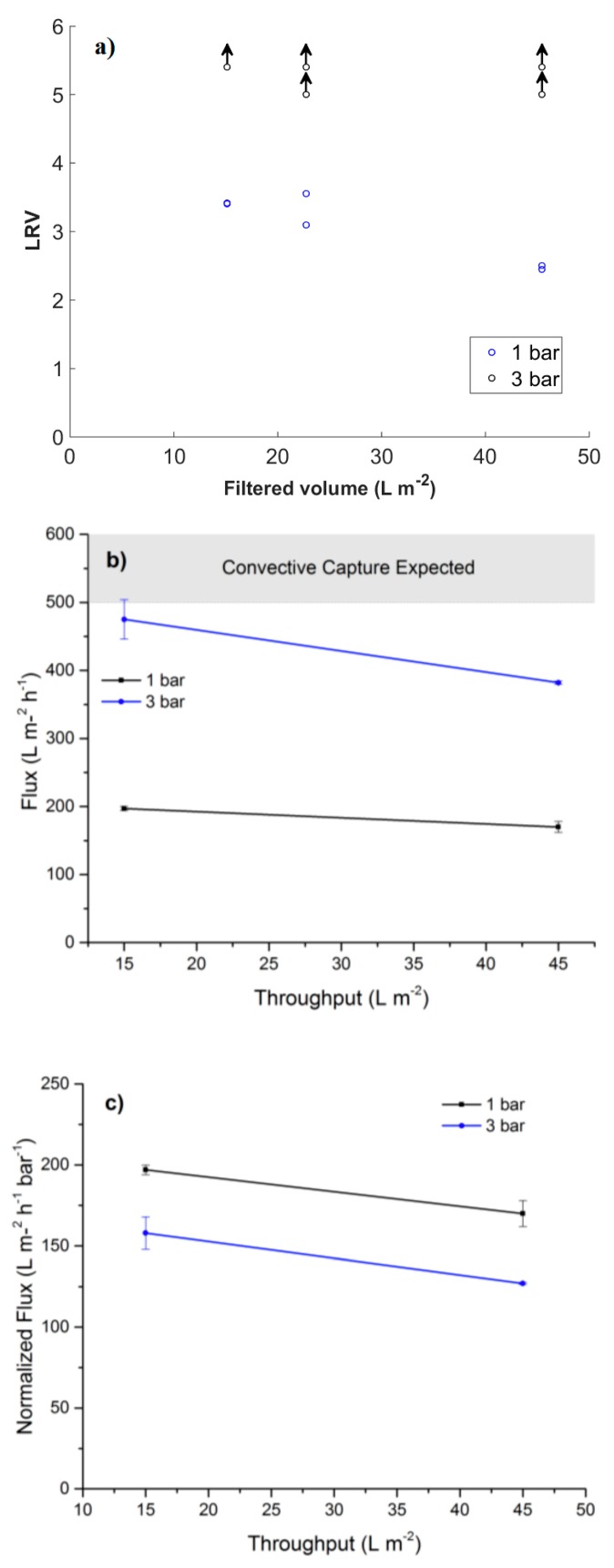
(**a**) Log reduction values (LRV) for filtration of ΦX174 bacteriophages at overhead pressures of 1 and 3 bar and a filter thickness of 9 µm. Arrows indicate that the obtained values are below the limit of detection (LOD). (**b**) Flux values for the filter at 1 and 3 bars, area marked in gray indicates fluxes where simulations predict hydrodynamic capture, i.e., Pe ≥ 1; For the 28 nm ΦX174 bacteriophages (**c**) Normalized flux per bar highlighting the compaction effect of the filter.

As seen in Figure 6, hydrodynamic constraint of gold particles is not expected at these fluxes. The same trend is observed for the ΦX174 bacteriophage, where u < ucr for fluxes ≤500 L h^−1^ m^−2^ in pores d ≤ 28 nm (marked by the vertical dashed line), therefore hydrodynamic constraint is not expected. An enhanced version of Figure 7 at *Pe* = 1 for the ΦX174 bacteriophages is available in the Supporting Information Figure S5.

### 3.4. Filtration of ΦX174 Bacteriophages

The LRVs, flux and normalized flux from filtration of ΦX174 bacteriophages are presented in Figure 7. The results show a clear difference in LRV for the two different pressures, where breakthrough of bacteriophages occurred for filtration at 1 bar. The retention of bacteriophage was also seen to decrease with filtered volume for filtration at 1 bar. For filtration at 3 bar, no bacteriophage was detected in permeate, and the two lower LRVs are due to a lower concentration of bacteriophage in the feed solution in the duplicate filtration of 45 L/m^2^ bacteriophage dispersion. The measured mean flux is presented in Figure 7b, only the initial flux value at 3 bar is close to the model predicted flux values where hydrodynamic capture is expected. For the 1 bar no hydrodynamic is expected according to the models. Neither 1 or 3 bar of pressure is enough to reach Pe ≥ 1, however, there is a distinct increase in LRV for filtration conducted at 3 bars. Figure 7c indicates filter compaction, which scales linearly with pressure which is a more probable explanation than hydrodynamic capture, filter compaction would limit lateral diffusion for the phages. The flux decay at higher throughput is probably due to removed phages blocking flow paths and fouling the filter.

### 3.5. Filtration of Nanoparticles

**Figure 8:**
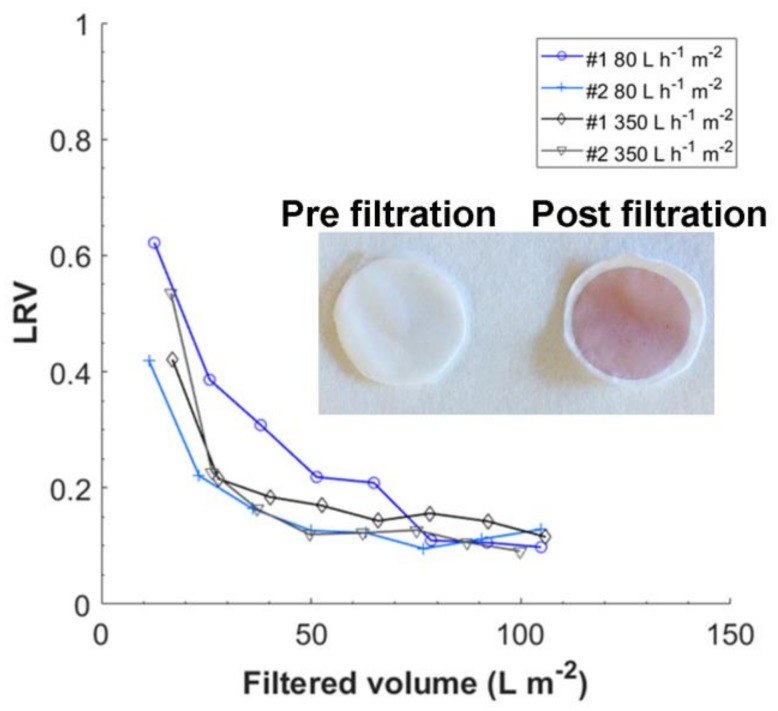
LRV for filtration of 5 nm gold particles at fluxes 80 and 350 L m^−2^ h^−1^. Inserted image shows a plain native unused filter pre and prost gold nanoparticle filtration.

In Figure 8 the results from filtration of 5 nm gold particles at fluxes 80 and 350 L·h^−1^·m^−2^ are shown. Also, 80 L·h^−1^·m^−2^ corresponds to a pressure of <2 bar while 350 L·h^−1^·m^−2^ corresponds to a pressure of around 3–4 bar.11 The results in Figure 8 do not indicate any influence of flux on the retention of 5 nm gold nanoparticles as seen in filtration of the bacteriophages. However, the gold nanoparticles where visibly trapped in the filter, as shown in the Figure 8 insert. An LRV of 0.5 for gold particles corresponds to a removal of 68% of all particles at the beginning of filtration. It should be noted that trapped the particles can easily be washed out during post-flush using the same buffer, see Supporting Information Figure S6. This suggests that the particles are rather hindered in the filter structure by weak association or due to the tortuous path than physically blocked.

In all, the theoretical simulations and experimental results of this work are helpful for understanding the virus removal filtration in nanocellulose-based filter paper in the context of bioprocessing of biological products. This is because viruses or prion particles (i.e., infectious misfolded proteins that lack DNA) are capable of self-replication under favorable conditions, unlike inorganic nanoparticles. Hence, breakthrough of infectious nanobioparticles during filtration even in limited numbers is a significant safety concern during the manufacturing of biological products.

## 4. Conclusions

In this study, the effect of Brownian motion in a nanocellulose-based virus removal filter paper was investigated through theoretical modeling and filtrations of gold nanoparticles and ΦX174 bacteriophages. Simulations of particle motion by solving the Langevin equation at different flow velocities reveal that constraint is favored for larger particles. The results from simulations were further confirmed by modeling of the Péclet number, indicating a greater contribution from convective forces, in contrast to Brownian forces, on particle motion for larger particles. Local flow velocities in pores have been modeled using two different approaches; solving of the Hagen-Poiseuille equation and evaluation of the superficial linear velocity. Filtration of 5 nm gold particles revealed no difference in retention of the investigated fluxes, which was predicted by both models for the local flow velocity, with regards to hydrodynamic constraint of particles. However, an unexpectedly high retention of as much as 68% of the particles was seen at the beginning of filtration. For filtration of ΦX174 bacteriophages, no breakthrough of phage was observed at a pressure of 3 bar, while phage breakthrough occurred at 1 bar. The observed difference in retentive behavior at different pressures for filtration of the ΦX174 bacteriophages was somewhat predicted by the Hagen-Poiseuille equation with regard to local flow velocities and hydrodynamic constraint. Local flow velocity modeling through superficial linear velocity did not predict any difference in retention for filtration at 1 and 3 bar. Overall, the hydrodynamic theory has been shown useful in explaining some of the observed flux-dependent retentive behaviors in virus filtration. However, limitations of the model with regards to electrostatic and chemical interparticle and particle-filter interactions, cumulative particle/virus load and filter thickness need to be highlighted. These areas might be subject to further studies. Also, cross-linking the cellulose nanofibers to limit the compaction of the filter at high pressure will be explored.

## Supplementary Materials

The following are available online at http://www.mdpi.com/2077-0375/8/4/90/s1, Figure S1: Brownian motion in two dimensions of three identical individual particles, Figure S2: Simulated particle trajectories for 5 nm gold particles in water at flow velocities, Figure S3: Simulated particle trajectories for 28 nm ΦX174 bacteriophages in water at flow velocities, Figure S4: Péclet number (Pe) as a function of the flow velocity (u) for 5 nm gold particles and 28 nm ΦX174 bacteriophages, Figure S5: Enhanced version of Figure 6 at Pe = 1 for the ΦX174 bacteriophages, Table S1: Measured thickness of nanocellulose filter papers, Table S2: Physical characteristics of particles used in Brownian hydrodynamic particle behavior simulations, Table S3: Physical parameters used in Brownian hydrodynamic particle behavior simulations.
