# Supplementary material for: Significance of Brownian Motion for Nanoparticle and Virus Capture in Nanocellulose-Based Filter Paper
Source: Membranes (Basel). 2018 Oct 5;8(4):90. doi: 10.3390/membranes8040090 (PMC6315380; doi:10.3390/membranes8040090)
Supplement: Supplementary file 1 [file membranes-08-00090-s001.pdf]

membranes

Supporting Information

Significance of Brownian Motion for Nanoparticle and Virus Capture in Nanocellulose-Based Filter Paper

Olof Gustafsson, Simon Gustafsson, Levon Manukyan and Albert Mihranyan *

Division of Nanotechnology and Functional Materials, Department of Engineering Sciences, Uppsala University, Box 534 SE-75121 Uppsala, Sweden; olof.gustafsson@kemi.uu.se (O.G.); simon.gustafsson@angstrom.uu.se (S.G.); levon.manukyan@angstrom.uu.se (L.M.)
* Correspondence: albert.mihranyan@angstrom.uu.se; Tel.: +46-18-471-7940

Received: 9 August 2018; Accepted: 25 September 2018; Published: 5 October 2018

INTRODUCTION

Early Observations of Pressure-Dependent Removal of Bioparticles. Already a century ago, it was argued that dialysis which is driven solely by molecular diffusion, *i.e.* at zero applied trans-membrane pressure, should be distinguished from ultrafiltration, which occurs due to pressure gradient across the filter [1]. As early as in 1905, Levy [2] performed ultrafiltration studies through applied pressure and compared the results to diffusion-driven dialysis for different enzymes. It was observed that several enzymes, e.g., ptyalin, renin, and pepsin, were able to pass through a nitrocellulose filter during dialysis. However, when these enzymes were then filtered through the same filter under applied pressure, no enzyme passed. In 1936, Elford and Ferry [1] reported a difference in the permeability of isoelectric serum albumin under different filtration conditions. Filtration through a membrane of 45 nm porosity at a pressure of 3 atm showed no permeability of the protein, whereas protein permeability was observed for pure diffusive filtration through a 14 nm porosity membrane. More recently, Asper [3] reported that virus breakthrough increased during filtration when pressure was released. These results were further confirmed by Zydney's group [4,5], who studied the retention of the ΦX174 bacteriophage in various industrial membranes during pressure release. Parvovirus breakthrough was detected at 1 bar but not at higher pressures, i.e. 2 or 3 bar, in Planova Bio-ex filters [6].

MATERIALS AND METHODS

Filtration of Gold Nanoparticles. The cellulose filters were placed in a stainless-steel syringe filter holder (13 mm, Millipore) with a Munktell General Purpose Filter Paper as mechanical support. The filters were wetted with 0.1 mM PBS solution (filtration of gold particles) prior to filtration for 5 minutes. Before the particle filtration, 0.8 mL of the buffer was pre-filtered to ensure that the entire filter structure was wetted.

A feed solution of 5 nm gold particles was prepared by diluting 1.25 mL of stock solution ($5.5 \cdot 10^{13}$ particles/mL) with 0.1 mM PBS aqueous solution up to a total volume of 25 mL, resulting in a concentration of $2.8 \cdot 10^{12}$ particles/mL.

Thickness Evaluation. The measured thickness of the nanocellulose papers is presented in table S1.

Table S1. Measured thickness of nanocellulose filter papers, i.e. five measurements per item at different locations.

Filter paper	Measured thickness (μm)
1	9, 8, 9, 9, 9
2	9, 11, 8, 9, 7
3	10, 9, 8, 9, 8
4	7, 7, 8, 8, 8
5	10, 9, 9, 9, 11

The resulting mean thickness of the nanocellulose filter papers is 9 μm (σ = 1 μm).

Filtration of ΦX174 Bacteriophages. A Luria-Bertani (LB) medium was prepared by mixing 10 g/L bactotryptone, 5 g/L bacto yeast extraction, and 10 g/L NaCl in deionized water. The pH was adjusted to pH 7.5 by adding 2.5 mL of 0.1 M NaOH. The buffer was then autoclaved at 121 °C for 20 minutes. A feed dispersion of ΦX174 bacteriophages was prepared by adding ΦX174 stock solution to Luria-Bertani (LB) medium to a resulting concentration of 10^6 phages/mL.

Feed and permeate solutions were serially diluted and then introduced to bacteria by adding 100 μL of sample to 200 μL of *E. coli* solution and mixing with 1 mL of soft agar (42 °C), which was then placed on hard agar plates. The plates were then incubated at 37 °C for 7 hours. After incubation, the number of visible plaques on the plates was observed, where one plaque corresponds to the occurrence of one bacteriophage, further on referred to as plaque forming unit (PFU). The bacteriophage concentration is hereby expressed as the number of PFU/mL and is calculated using equation S1.

$$log_{10}\left(\frac{PFU}{ml}\right) = log_{10}\left(\frac{number\ of\ plaques}{0.1 \cdot dilution\ factor}\right) \quad (S1)$$

The value 0.1 corresponds to the volume of bacteriophage solution on each plate. Bacteriophage removal rate is described in terms of LRV and is subsequently calculated using equation S2.

$$LRV = log_{10}\left(\frac{PFU}{ml}\right)_{feed} - log_{10}\left(\frac{PFU}{ml}\right)_{permeate} \quad (S2)$$

The occurrence of any bacteriophage in the remaining permeate solution was controlled through so-called large volume plating (LVP). 5 mL of *E. coli* solution was added to permeate solutions before incubating at 37°C at 120 rpm for 7 hours. *E. coli* added to LB medium and incubated at the same conditions was used as a bacteriophage-free reference. After incubation, the optical density was measured at 600 nm for permeates and references.

Nitrogen Sorption Measurements

The pore size distribution of the nanocellulose filter papers was evaluated with the Barret-Joyner-Halenda (BJH) method using the desorption branch of the isotherm [7]. This was done using an ASAP 2020 (Micrometrics, USA) instrument. The filter sample was degassed at 90 °C in vacuum for four hours and the following analysis of nitrogen sorption was then carried out at 77 K using liquid nitrogen as a coolant.

RESULTS AND DISCUSSION

Brownian Motion in a Flowing Fluid. The direction of motion the particle undergoes is completely random. However, the magnitude of the motion is dependent on the particle size, viscosity of the liquid, temperature and time. The mathematical expression for Brownian motion is shown in equation S3 [8,9].

$$\langle x^2\rangle^{1/2} = (2Dt)^{1/2} \quad \text{(S3)}$$

where $\langle x^2\rangle^{1/2}$ is the root mean square distance, which describes the average mean squared displacement of a particle at time t. D is the diffusion constant, further expressed in equation S4 [8].

$$D = \frac{k_B T}{3\pi\eta d_p} \quad \text{(S4)}$$

where kB is the Boltzmann constant, T is the temperature, η is the dynamic viscosity of the liquid and dp is the particle diameter.

Since the nature of Brownian motion is random, the distance traveled by a specific particle can be both greater and smaller than the average mean square displacement, as it is merely the average derived from a great number of particles. The average mean square displacement is also independent of direction and only states the displacement in absolute numbers.

Figure S1 illustrates Brownian motion in two dimensions for three identical individual particles starting at (0,0). As seen in figure S1 the motion of the particles differs in space, but the distance traveled from the starting point is on the same scale.

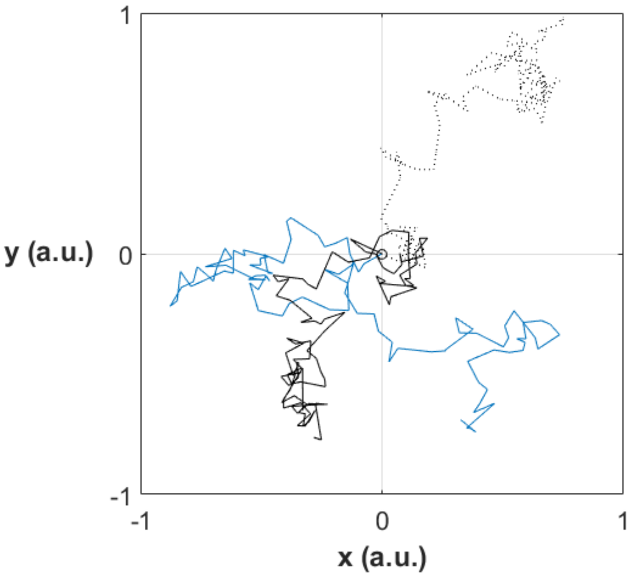

Figure S1. Brownian motion in two dimensions of three identical individual particles. Each line represents the trajectory of an individual particle and all particles start at (0,0).

The Langevin equation relates Brownian forces to viscous forces on a particle moving in a liquid as shown below: [10–12]

$$\frac{dv}{dt} = -\zeta'(v - v_f) + \frac{1}{m'}F^B \quad \text{(S5)}$$

The first part of equation S5 represents the contribution of the viscous forces on the movement of the particle and is derived from the Stokes' law which was proposed by George Gabriel Stokes in 1851 [13]. v is the particle velocity, v_f is the velocity of the fluid and ζ' is a term further expressed in equation S6.

$$\zeta' = \frac{3\pi\eta d_p}{m'} \quad \text{(S6)}$$

where η is the dynamic viscosity of the fluid, d_p is the particle diameter and m' is the apparent mass of the particle, which is described in equation S7.

$$m' = m + \frac{\pi (d_p)^3 \rho_{fluid}}{12} \qquad \text{(S7)}$$

m is the mass of the particle and ρ_{fluid} is the density of the fluid.

The term added to the particle mass in equation S7 is also known as the virtual mass or added mass, and was proposed by Odar and Hamilton in 1964 [14] and verified by Odar in 1966 [15] for spheres moving in a fluid. The added mass accounts for the resisting force that is induced on a sphere moving through a viscous fluid as a result of a change in the kinetic energy of the displaced fluid. The added mass, m_{added}, is expressed in equation S8 [14].

$$m_{added} = C_A \frac{4\pi r_{sphere}^3 \rho_{fluid}}{3} \qquad \text{(S8)}$$

Where C_A is the added mass coefficient, r_{sphere} is the radius of the sphere and ρ_{fluid} is the density of the fluid. Milne-Thomson further theorized the concept in 1968 [16] for spheres specifically, with the resulting added mass coefficient, C_A, for spheres being equal to 1/2.

The second part of equation S5 represents the contribution of random forces due to Brownian motion, and these are expressed in the term F^B.

If equation S5 is integrated, expressions for particle velocity, v, and particle position, r, are given as shown in equations S9 and S10 [11,12].

$$v(t + \Delta t) - v(t) = -(v(t) - v_{solvent})\left(1 - e^{-\zeta' \Delta t}\right) + \delta v^B \qquad \text{(S9)}$$

$$r(t + \Delta t) - r(t) = \frac{1}{\zeta'}(v(t) - v_{solvent})\left(1 - e^{-\zeta' \Delta t}\right) + v_{solvent}\Delta t + \delta r^B \qquad \text{(S10)}$$

Velocity and position at a certain time t are expressed as $v(t)$ and $r(t)$ and the subsequent expressions at a time $(t+\Delta t)$ are $v(t+\Delta t)$ and $r(t+\Delta t)$. The two terms δv^B and δr^B represent the contribution from Brownian motion and can be determined stochastically.

Stochastic Modeling of Brownian Motion. If movement in two dimensions is considered, δv^B and δr^B can be written as $\delta v^B(v_1,v_2)$ and $\delta r^B(r_1,r_2)$, where v_i and r_i are random numbers sampled from the probability density function stated in equation S11 [11,12].

$$\rho(r_i, v_i) = \frac{1}{2\pi\sigma_r\sigma_v(1 - c_{rv}^2)^{1/2}} \cdot exp\left[-\frac{1}{2(1 - c_{rv}^2)}\left\{\left(\frac{r_i}{\sigma_r}\right)^2 - 2c_{rv}\frac{r_i}{\sigma_r}\frac{v_i - \bar{v}_i}{\sigma_v} + \left(\frac{v_i - \bar{v}_i}{\sigma_v}\right)^2\right\}\right] \qquad \text{(S11)}$$

The terms σ_r^2, σ_v^2 and c_{rv} are expressed in equations S12-S14.

$$\sigma_r^2 = \frac{k_B T}{m' \zeta'^2}\left(2\zeta'\Delta t - 3 + 4e^{-\zeta'\Delta t} - e^{-2\zeta'\Delta t}\right) \qquad \text{(S12)}$$

$$\sigma_v^2 = \frac{k_B T}{m'}\left(1 - e^{-2\zeta'\Delta t}\right) \qquad \text{(S13)}$$

$$c_{rv} = \frac{1}{\sigma_r \sigma_v}\frac{k_B T}{m' \zeta'}\left(1 - e^{-\zeta'\Delta t}\right)^2 \qquad \text{(S14)}$$

Where k_B is the Boltzmann constant and T is the temperature. Using inverse transform sampling on equation S11, the expressions for v_i and r_i condense to equation S15 and S16 [11].

$$v_i = \left(-2\sigma_v^2 lnR_{i,1}\right)^{1/2} cos2\pi R_{i,2} \qquad \text{(S15)}$$

$$r_i = c_{rv}\frac{\sigma_r}{\sigma_v}v_i + (1 - c_{rv}^2)^{1/2}\left(-2\sigma_v^2 lnR_{i,3}\right)^{1/2} cos2\pi R_{i,4} \qquad \text{(S16)}$$

$R_{i,1}$, $R_{i,2}$, $R_{i,3}$ and $R_{i,4}$ are random numbers from the uniform stochastic distribution (0,1).

Theoretical Modeling of Hydrodynamic Velocity and Brownian Motion. The physical characteristics of the gold nanoparticles and ΦX174 bacteriophages used in the simulations can be found in table S2.

Table S2. Physical characteristics of particles used in Brownian hydrodynamic particle behavior simulations.

Particle	Diameter (nm)	Molecular weight (kDa)	Particle mass (kg)
Au	5 [a]	-	$1.27 \cdot 10^{-21}$ [a]
ΦX174	28 [17]	6 200 [18]	$1.03 \cdot 10^{-20}$

[a] – Information as supplied by manufacturer.

The remaining physical parameters used in the simulations are summed up in table S3. The choice of Δt was validated through equation S3, which gives the mean squared displacement over time, and Δt was chosen so that the simulated motions where on the same order of magnitude as the mean squared displacement calculated from equation S3. Convergence in the simulation was considered to have been reached in this case.

Table S32. Physical parameters used in Brownian hydrodynamic particle behavior simulations.

Parameter	Value
t	$1 \cdot 10^{-4}$ s
Δt	$(1 \cdot 10^{-4}/300)$ s
ρ_{fluid}, water	$1.0 \cdot 10^{3}$ kg/m^3
η, water	$1.002 \cdot 10^{-3}$ Pa·s
T	293 K
k_B	$1.38 \cdot 10^{-23}$ J/K

The results from simulations of flow velocity effect on the Brownian motion of 5 nm gold particles at flow velocities $1 \cdot 10^{-5}$ m/s, $1 \cdot 10^{-3}$ m/s, $2 \cdot 10^{-2}$ m/s and $5 \cdot 10^{-2}$ m/s are shown in figure S2a-d. The results from simulations of flow velocity effect on the Brownian motion of 28 nm ΦX174 bacteriophages at flow velocities $1 \cdot 10^{-5}$ m/s, $1 \cdot 10^{-3}$ m/s, $2 \cdot 10^{-2}$ m/s and $5 \cdot 10^{-2}$ m/s are shown in figure S3a-d.

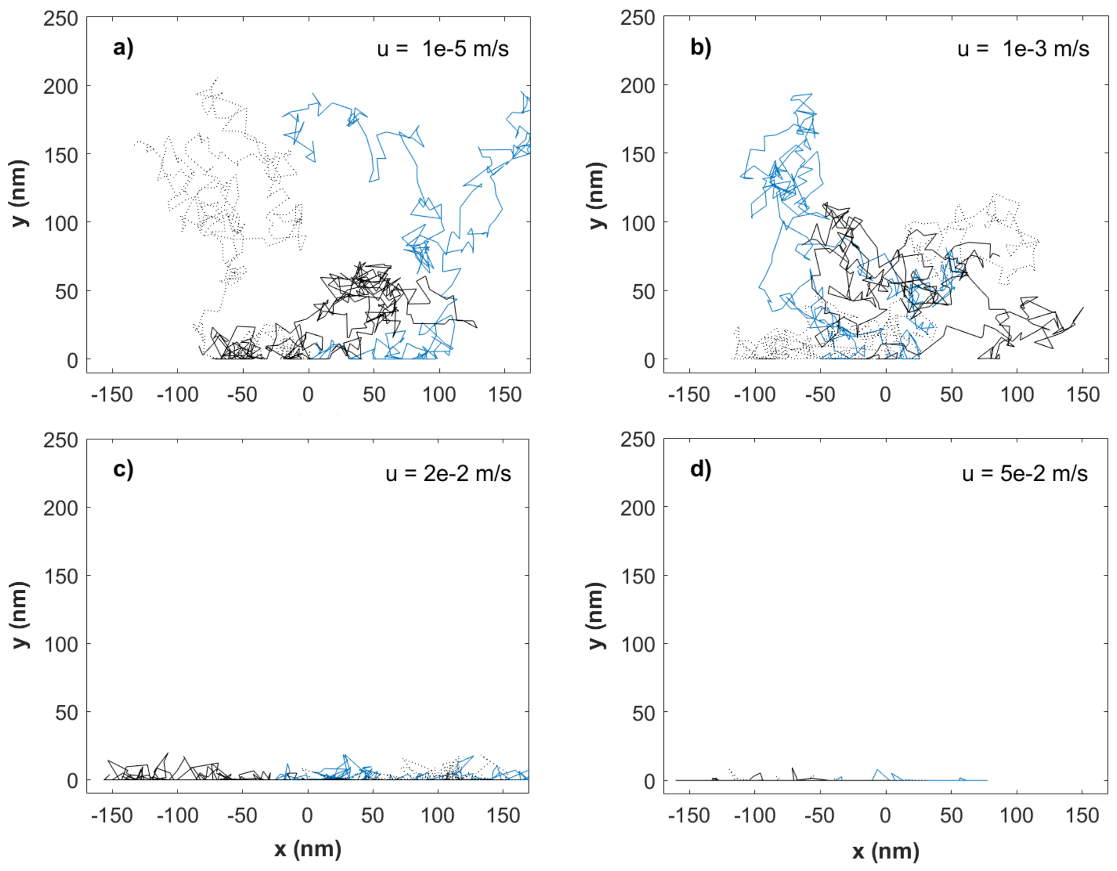

Figure S2. Simulated particle trajectories for 5 nm gold particles in water at flow velocities a) $1{\cdot}10^{-5}$ m/s, b) $1{\cdot}10^{-3}$ m/s, c) $2{\cdot}10^{-2}$ m/s and d) $5{\cdot}10^{-2}$ m/s. Each line represents one particle trajectory.

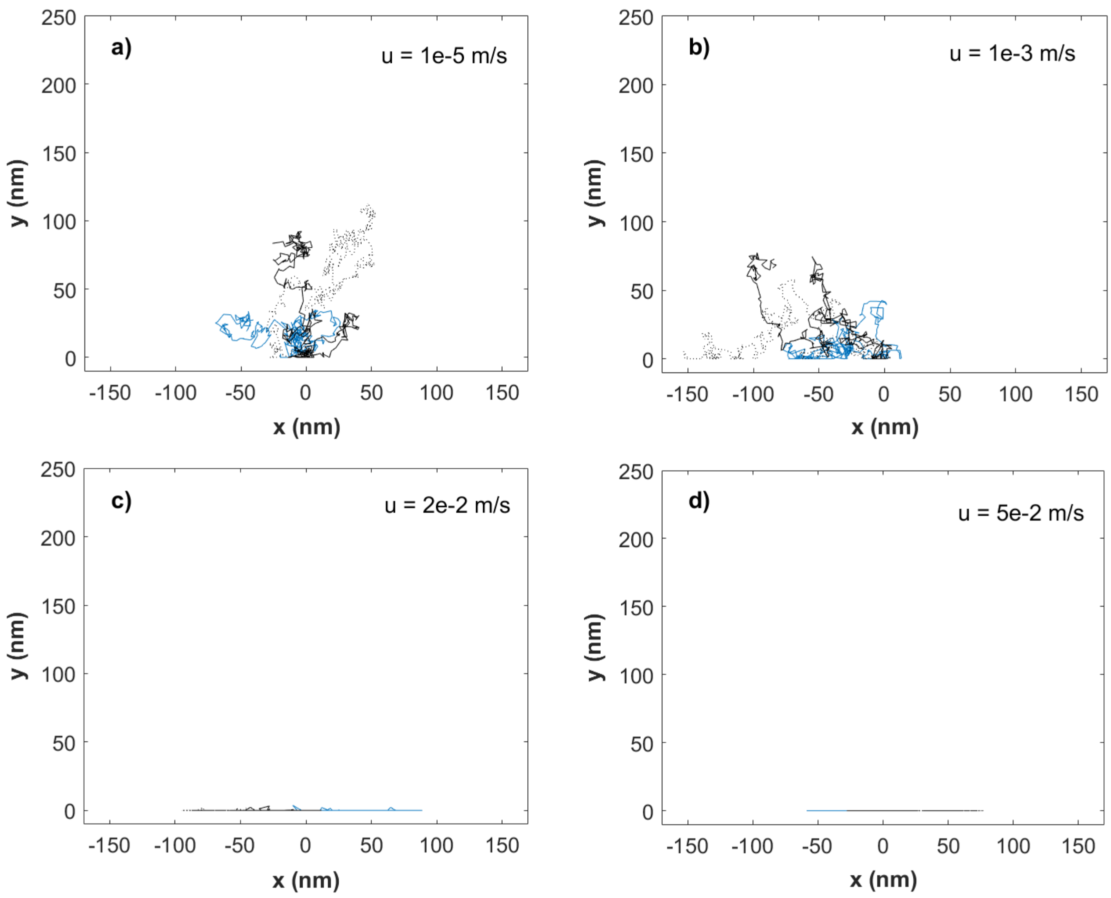

Figure S3. Simulated particle trajectories for 28 nm ΦX174 bacteriophages in water at flow velocities a) $1{\cdot}10^{-5}$ m/s, b) $1{\cdot}10^{-3}$ m/s, c) $2{\cdot}10^{-2}$ m/s and d) $5{\cdot}10^{-2}$ m/s. Each line represents one particle trajectory.

The Péclet Number. As previously described by Trilisky and Lenhoff [19], u_{cr} is defined as the flow velocity where the contribution from the Brownian forces on particle motion is overcome by the convective forces from the flow. For the simulations in figure 1, u_{cr} appears to be somewhere in the region of $1{\cdot}10^{-2}$ m/s, as there is a noticeable effect of hydrodynamic constraint on both particle types at this flow velocity. The concept of a critical velocity can be further evaluated by investigating the Péclet number (*Pe*), expressed in equation S17 [19].

$$Pe = \frac{ud_p}{D} \qquad (S17)$$

Where u is the flow velocity, d_p is the diameter of the particle and D is the diffusion constant. With the full expression for the diffusion constant D, equation S17 can be rewritten as equation S18.

$$Pe = \frac{3\pi\eta u d_p^2}{k_B T} \qquad (S18)$$

In figure S4, the Péclet number is shown as a function of flow velocity for both 5 nm gold particles and 28 nm ΦX174 bacteriophages. The required flow velocity for convection dominated motion of particles, *i.e.* $Pe > 1$, is lower for the ΦX174 bacteriophages as compared to the gold particles.

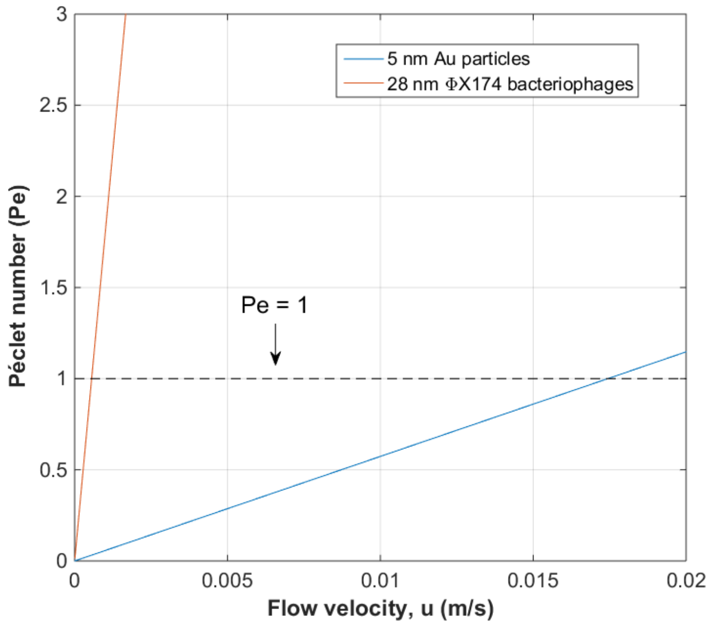

Figure S4. Péclet number (*Pe*) as a function of the flow velocity (*u*) for 5 nm gold particles and 28 nm ΦX174 bacteriophages.

Modeling of Local Flow Velocities. A basis for the validity of the Hagen-Poiseuille equation is that the flow is laminar, *i.e.* a Reynolds number smaller than 2300 [20]. Reynolds number (*Re*) is further expressed in equation S19 [20].

$$Re = \frac{uD}{\eta} \qquad (S19)$$

where u is the flow velocity and D is the capillary diameter. For the nanocellulose-based virus removal filter paper in this study, nitrogen sorption measurements revealed a BJH desorption pore-size distribution of pore widths 3-46 nm with a peak pore width of 23 nm, as seen in figure 1. The critical flow velocity for turbulent flow of water, *i.e.* $Re = 2300$, through capillaries with a width of 23 nm, is $1\cdot10^{8}$ m/s as calculated from equation S19. Thus, the flow is considered as being laminar.

An enhanced version of figure 6 can be found in figure S5. Local flow velocity u as a function of pore width d is shown around $Pe = 1$ for the ΦX174 bacteriophage.

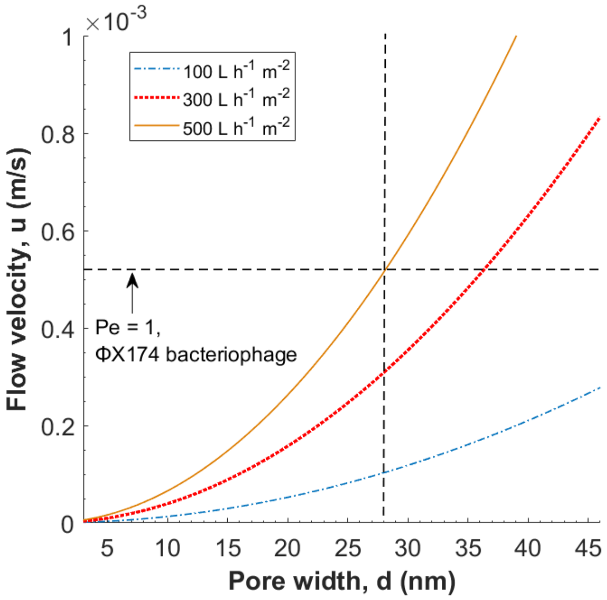

Figure S5. Enhanced version of figure 6 at $Pe = 1$ for the ΦX174 bacteriophages. Local flow velocity u in pores of width 3-46 nm and d_m = 23 nm, at fluxes 100, 300 and 500 L h^{-1} m^{-2}. Figure constructed using equation 3. Horizontal dashed lines indicate $Pe = 1$, *i.e.* $u = u_{cr}$, for ΦX174 bacteriophages. The vertical dashed line at d = 28 nm highlights a pore width equal to the diameter of the ΦX174 bacteriophage.

Post-flushing of Filters after Nanoparticle Filtration. Post-flush experiments were carried out for filtrations of 5 nm gold particles. Filtrations were performed as previously explained in this work. After filtration of gold nanoparticles, the filters were flushed with 0.1 mM PBS solution at the same flux settings and the permeate solution was collected for further measuring of absorbance. The absorbance was translated to concentration, using equation S20.

$$Concentration = \frac{AUC_{permeate}}{1.73 \cdot 10^{-12}} \qquad (S20)$$

Where the concentration is given in particles/mL, $AUC_{permeate}$ is the area under the curve for the permeate and $1.73 \cdot 10^{-12}$ is given as the slope of the calibration curve for 5 nm gold particles. The result from post-flush at flux 80 L h^{-1} m^{-2} is shown in figure S6.

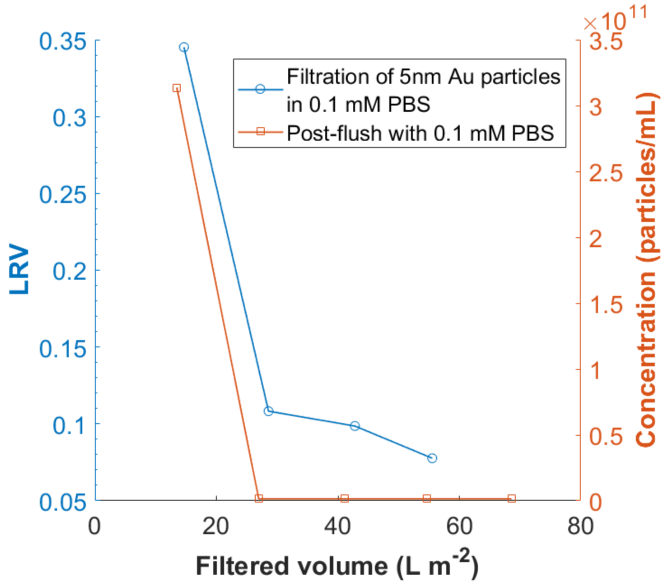

Figure S6. Log reduction value (LRV) and permeate concentration for filtration of 5 nm gold particles and consecutive post-wash with 0.1 mM PBS at a flux of 80 L h^{-1} m^{-2}.

As seen in figure S6, at a flushed volume of 13 L m^{-2}, gold particles were detected in the permeate solution. At flushed volumes ≥27 L m^{-2}, no gold particles were detected in the permeate solution. After flushing, there were no particles visibly trapped in the filter, as opposed to that shown in figure 9a.

BJH Pore Size Measurement

Figure S7 present the pore size distribution of an 11 μm thick filter. The nominal pore size distribution is centered at 23 nm.

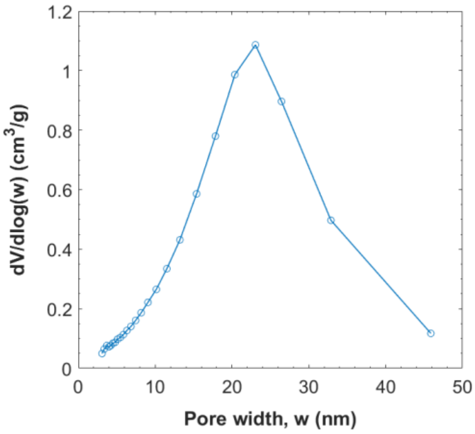

Figure S7. BJH N2 gas desorption pore size distribution of the nanocellulose filter paper. The pore mode of the filter is 23 nm

References

1. Ferry, J.D. Ultrafilter Membranes and Ultrafiltration. *Chemical Reviews* **1936**, *18*, 373-455, doi:10.1021/cr60061a001.
2. Levy, D.J. Some Physical Properties of Enzymes. *The Journal of Infectious Diseases* **1905**, *2*, 1-48, doi:10.1093/infdis/2.1.1.
3. Asper, M. Virus breakthrough after pressure release during virus retentive filtration. In Proceedings of PDA, Barcelona, Spain, 2011.
4. Woods, M.A.; Zydney, A.L. Effects of a Pressure Release on Virus Retention with the Ultipor DV20 Membrane. *Biotechnology and Bioengineering* **2014**, *111*, 545-551, doi:10.1002/bit.25112.
5. Dishari, S.K.; Venkiteshwaran, A.; Zydney, A.L. Probing Effects of Pressure Release on Virus Capture During Virus Filtration Using Confocal Microscopy. *Biotechnology and Bioengineering* **2015**, *112*, 2115-2122, doi:10.1002/bit.25614.
6. Chen, D. Viral Clearance Using Traditional, Well-Understood Unit Operations (Session I): Virus-Retentive Filtration. *PDA Journal of Pharmaceutical Science and Technology* **2014**, *68*, 38-50, doi:10.5731/pdajpst.2014.00965.
7. Barrett, E.P.; Joyner, L.G.; Halenda, P.P. The Determination of Pore Volume and Area Distributions in Porous Substances. I. Computations from Nitrogen Isotherms. *J. Am. Chem. Soc.* **1951**, *73*, 373-380, doi:10.1021/ja01145a126.
8. Nelson, E. Dynamical Theories of Brownian Motion. *Mathematical Notes* **1967**, *131*, 2381-2396, doi:10.1103/PhysRev.131.2381.
9. Atkins, P.; de Paula, J. *Atkins' Physical Chemistry*; Oxford University Press: Oxford, 2010; pp. 773; 9780199543373.
10. Lemons, D.S.; Gythiel, A. Paul Langevin's 1908 paper "On the Theory of Brownian Motion" ["Sur la Théorie du Mouvement Brownien," C. R. Acad. Sci. (Paris) 146, 530–533 (1908)]. *American Journal of Physics* **1997**, *65*, 1079-1081, doi:10.1119/1.18725.
11. Satoh, A. *Introduction to Molecular-Microsimulation of Colloidal Dispersions*; Elsevier: Amsterdam, 2003; 10.1016/j.jcis.2003.08.070pp. 127-132; 0444514244.
12. Yamamoto, A.; Hongo-Hirasaki, T.; Uchi, Y.; Hayashida, H.; Nagoya, F. Effect of Hydrodynamic Forces on Virus Removal Capability of Planova™ Filters. *Aiche Journal* **2014**, *60*, 2286-2297, doi:10.1002/aic.14392.
13. Stokes, G.G. On the Effect of the Internal Friction of Fluids on the Motion of Pendulums. *Transactions of the Cambridge Philosophical Society* **1851**, *9*, 8-8.
14. Odar, F.; Hamilton, W.S. Forces on a Sphere Accelerating in a Viscous Fluid. *Journal of Fluid Mechanics* **1964**, *18*, 302-314, doi:10.1017/s0022112064000210.
15. Odar, F. Verification of the Proposed Equation for Calculation of the Forces on a Sphere Accelerating in a Viscous Fluid. *Journal of Fluid Mechanics* **1966**, *25*, 591-592, doi:10.1017/s0022112066000272.

16. Milne-Thomson, L.M. *Theoretical Hydrodynamics*; Macmillan: London, 1968; 9780333078761.
17. Liu, S.; Carroll, M.; Iverson, R.; Valera, C.; Vennari, J.; Turco, K.; Piper, R.; Kiss, R.; Lutz, H. Development and Qualification of a Novel Virus Removal Filter for Cell Culture Applications. *Biotechnology Progress* **2000**, *16*, 425-434, doi:10.1021/bp000027m.
18. Sinsheimer, R.L. Bacteriophage ϕX174. King, R.C., Ed. Springer US: Boston, MA, 1974; pp. 323-325; 9781489917102.
19. Trilisky, E.I.; Lenhoff, A.M. Flow-Dependent Entrapment of Large Bioparticles in Porous Process Media. *Biotechnology and Bioengineering* **2009**, *104*, 127-133, doi:10.1002/bit.22370.
20. Rott, N. Note on the History of the Reynolds Number. *Annual Review of Fluid Mechanics* **1990**, *22*, 1-12, doi:10.1146/annurev.fl.22.010190.000245.

© 2018 by the authors. Submitted for possible open access publication under the terms and conditions of the Creative Commons Attribution (CC BY) license (http://creativecommons.org/licenses/by/4.0/).
